# Workplace Violence and Mental Wellbeing Among Long-Term Care Nursing Assistants in Different Work Locations: A Cross-Sectional Study

**DOI:** 10.1155/jonm/7817632

**Published:** 2025-02-28

**Authors:** Hang-Ju Yang, Yen-Ling Liu, Li-Chung Pien, Yun-Chieh Yang, Wan-Ju Cheng

**Affiliations:** ^1^Department of Emergency Medicine, Jen Ai Hospital Dali Branch, 483 Dong Rong Road, Dali, Taichung, Taiwan; ^2^School of Nursing, China Medical University, 100 Section 1, Jingmao Road, Taichung 406040, Taiwan; ^3^Post-Baccalaureate Program in Nursing, College of Nursing, Taipei Medical University, 250 Wuxing Street, Taipei 11031, Taiwan; ^4^Psychiatric Research Center, Wan Fang Hospital, Taipei Medical University, Taipei 116079, Taiwan; ^5^National Center for Geriatrics and Welfare Research, National Health Research Institutes, 35 Keyan Road, Miaoli, Taiwan; ^6^Department of Public Health, China Medical University, 100 Section 1, Jingmao Road, Taichung 706040, Taiwan; ^7^Department of Psychiatry, China Medical University Hospital, 2 Yude Road, Taichung 404327, Taiwan

**Keywords:** ageing, burnout, caregiver, home, psychological, work conditions

## Abstract

**Aims:** This study aims to investigate the association between workplace violence and mental wellbeing of long-term care nursing assistants (LTC-NAs) based on work locations.

**Background:** The increasing global elderly population is elevating the demand for LTC services. The LTC-NA population is expanding, highlighting the necessity to create a secure work environment. However, little is known about how workplace violence poses a threat to the mental wellbeing of LTC-NAs across different work locations.

**Methods:** This observational cross-sectional study was conducted from October 2022 to July 2023, involving a survey of 937 certified LTC-NAs recruited through convenience sampling from various Taiwanese LTC units. Participants were evaluated for experiences of four types of workplace violence (physical, psychological, verbal, and sexual harassment) using a disseminated questionnaire. Mental health was assessed using the 5-item Brief Symptom Rating Scale, and client-related burnout was evaluated with the 6-item scale from the Chinese version of Copenhagen Burnout Inventory. Logistic regression identified the association between workplace violence and mental wellbeing. In addition, the relationship among participants working in residential facilities or home settings were examined.

**Results:** The completion rate of the questionnaire was 86%. Psychological violence was associated with poor mental health (adjusted odds ratio [OR] = 2.38 and 95% CI = 1.40–4.05), while verbal violence and sexual harassment were associated with client-related burnout (adjusted OR = 2.03 and 1.75, respectively). All types of workplace violence were more prevalent in residential facilities than home settings; however, the associations of workplace violence with poor mental wellbeing were more prominent among those working in home settings. Among violence victims, a higher proportion of LTC-NAs working in home settings reported experiencing physical and psychological violence from patients' families compared to those working in residential facilities. Client-related burnout was found to mediate the relationship between violence from patients' families and poor mental health.

**Conclusion:** Nonphysical workplace violence and sexual harassment were associated with poor mental wellbeing among LTC-NAs, especially in homecare settings. Violence from patients' families posed a notable risk to homecare LTC-NAs. Therefore, protective policies and organizational training programs should be tailored to address the unique challenges of each work setting.

## 1. Introduction

The global phenomenon of an aging society, coupled with shifts in family demographics, has intensified the caregiving burden, resulting in a significant rise in the demand for long-term care (LTC) services. In Taiwan, by the end of 2022, there were over 90,000 LTC nursing assistants (LTC-NAs); however, there is still a shortage to meet the demand [1]. Establishing a positive work environment is crucial for maintaining a stable LTC-NA workforce and their wellbeing, as workplace violence poses a significant threat [2, 3].

Workplace violence is defined as any incident in which employees are abused, threatened, or assaulted in connection with their work [4, 5]. Four main types of workplace violence have been identified: physical violence (e.g., hitting, biting, and kicking), verbal abuse (e.g., insults, yelling, and sarcasm), psychological violence (e.g., threats, harassment, and intimidation), and sexual harassment (e.g., inappropriate sexual advances), all of which impact individuals' safety, mental wellbeing, and health [4, 6]. Workplace violence may be committed by patients, colleagues, patients' family members, or management [6]. A systematic review of global workplace violence rates among healthcare workers revealed that 62% of healthcare workers have encountered incidents of workplace violence [7]. A study conducted in nursing homes estimated that 34% of LTC-NAs employed experienced physical aggressions in the past year [8]. Workplace violence poses a significant threat to workers' mental health. Those who have experienced workplace violence are more likely to experience burnout, posttraumatic stress disorder, psychological distress, anxiety, and depression among nurses and homecare workers [9, 10]. In Taiwan, LTC-NAs are frontline workers who directly interact with individuals requiring LTC services. A report by the Ministry of Labor of Taiwan revealed that verbal abuse (36.89%) was the most common form of workplace violence, while sexual harassment was more prevalent in home care (25.93%), posing risks to the mental wellbeing LTC-NAs [11]. Nevertheless, qualitative studies revealed that in the context of LTC, workers often banalize workplace violence [3, 12], and the perception of abuse was moderated by service recipients' illness such as dementia [13].

Most studies have focused on workplace violence within the context of LTC residential facilities [12–14]. In contrast, home care, as opposed to hospitalization and LTC residential facilities, offers a cost-effective alternative for individuals residing in rural areas or lacking family support [15]. However, home-care healthcare workers, particularly paraprofessionals, including LTC-NAs, are observed to be at risk of nonphysical aggression [16], sexual violence, and sexual harassment [17]. Working in clients' homes exposes LTC-NAs to a relatively uncontrolled work environment [18], performing their job in isolation [19], and in the absence of employer safety policies and protective settings [20]. While home LTC-NAs often have to deal with abuse and violence by themselves [17], the impact of workplace violence on their mental wellbeing has been less studied.

Compared with physical violence, nonphysical forms, such as verbal and psychological violence, are more frequently experienced in healthcare workplace [21]. Internal workplace violence, occurring between workers and their colleagues or supervisors, has a more significant impact on mental health than external violence, where violence sources are patients and their families [22]. The prevalence of different types and sources of workplace violence varies by work locations, such as clients' home or hospitals, and they impact workers' health differently [2, 9]. However, limited research has explored the interaction between work location and the type or source of violence.

In Taiwan, nearly two-thirds of LTC-NAs perform home-based work [1]. However, most literature on violence experienced by LTC-NAs is focused on those working in geriatric facilities and nursing homes [8, 12]. While home-based LTC-NAs often experience abuse and violence in isolation [17], the impact on their mental wellbeing has been less studied. Furthermore, limited research has explored how work location influences the type or source of violence. Studies examining different types of workplace violence have primarily focused on hospital nurses [8, 12, 22–24], with limited attention given to LTC-NAs. Therefore, workplace violence by LTC-NAs requires further investigation. This study aims to (1) investigate the association between different types of workplace violence and mental wellbeing among LTC-NAs and (2) examine the prevalence of workplace violence in different work locations, as well as the differential association with mental wellbeing.

## 2. Materials and Methods

### 2.1. Study Design

This cross-sectional survey employed convenience sampling to recruit LTC-NAs aged 20 and older from healthcare and LTC settings in Taiwan, encompassing hospitals, nursing homes, and home-based LTC units. Taiwan's population is rapidly aging, with individuals aged 65 and older comprising 18% of the total population. In 2023, an estimated 704,999 individuals required LTC services, while 91,653 LTC-NAs were registered. We calculated the required sample size for a survey, determining that, with a 95% confidence interval and a 5% margin of error, a minimum of 383 participants was required. To account for an estimated 20% rate of invalid responses, the final target sample size was set at 479 participants. Data were collected through both online and paper questionnaires. Online surveys were administered via social media platforms and communication apps, while paper questionnaires were distributed during in-service training courses and unit supervisory meetings. In both cases, participants were informed that they were invited to take part in the study anonymously, and they received a 50 New Taiwan dollar voucher as a monetary reward. The study received review and approval from the Institutional Review Board (CMUH111-REC2-165).

### 2.2. Study Participants

We recruited 1094 certified LTC-NAs, specifically those who had completed the training course mandated by the LTC Services Act and obtained certification from the Ministry of Health and Welfare as recognized LTC personnel. All participants were employed at the time of the survey. We excluded participants who reported having less than 3 months of working experience as an LTC-NA (*N* = 55) and those who self-reported diagnosis of depression, bipolar disorder, anxiety, or panic disorder (*N* = 65). Furthermore, 3 participants were excluded due to missing burnout data, 10 due to missing BSRS scores, and 24 due to missing control variables. Ultimately, 937 participants were included in the final analysis, with a questionnaire completion rate of 86%.

### 2.3. Measurements

#### 2.3.1. Workplace Violence

Participants were surveyed regarding their experiences of four types of workplace violence in the past 12 months: physical violence (e.g., hitting, kicking, pushing, and pulling), verbal violence (e.g., insults, verbal harassment, and sarcasm), psychological violence (e.g., threats, intimidation, discrimination, exclusion, bullying, and harassment), and sexual harassment (e.g., inappropriate sexual innuendos and behavior). Each participant's response was recorded as “yes” or “no.” In addition, participants were asked to identify the source of each type of violence, which was categorized into three groups: patients, patients' family, and colleagues or supervisors.

#### 2.3.2. Work Conditions and Control Variables

The participants selected their work locations from nursing homes, hospitals, and home-based LTC units. Work locations were categorized into two groups: home settings and residential facilities, which include nursing homes and hospitals. Work conditions included work tenure, job control, and psychological job demands. Work tenure was categorized into three groups: 1 year or lower, 2-3 years, and 4 years or above. Job control and psychological job demands were assessed using the validated Chinese version of the Job Content Questionnaire (C-JCQ) based on the job strain model by Karasek and Theorell [25, 26]. A 7-item questionnaire for the demands scale (work is fast, work is hectic, work is hard, workloads are excessive, sufficient time for the job, must concentrate on the job for a long time, and workplace understaffed) and a 9-item questionnaire for the control scale (learning new things, nonrepetitive work, creative work, high-skill work, various tasks, can develop one's abilities, allowed to make own decisions, freedom to make decision, and opinion is influential) were used.

Sex, age, marital status, education level, and annual income were self-reported. Education level was categorized into two groups: secondary or lower and university or above. Marital status was categorized as married or other status (separated, divorced, widowed, or single). Participants reported their annual income in relation to their roles as LTC-NAs by selecting predefined categories. We grouped participants into high and low-income categories using a New Taiwan dollar threshold of 500,000 per year (equivalent to approximately 15,514 U.S. Dollars).

#### 2.3.3. Outcome Measurements

The outcome measures included mental wellbeing assessed by the Brief Symptom Rating Scale (BSRS-5) and client-related burnout. The BSRS-5 assessed mood changes over the past week and demonstrated good validity and reliability across different populations, with Cronbach's *α* ranging from 0.77 to 0.90 in previous study of Taiwanese hospitalized patients [27]. Each item in the BSRS-5 was assigned scores ranging from 0 to 4. The total score ranged from 0 to 20, with higher scores indicating poorer mental health. Participants with total BSRS-5 scores of six or more were defined as having poor mental health.

Client-related burnout was assessed by the 6-item scale from the Chinese version of Copenhagen Burnout Inventory, with Cronbach's *α* ranging from 0.90 to 0.91 in Taiwanese workers [28], which has demonstrated good reliability and validity and is widely employed to evaluate employees' health status among employees. Participants provided ratings for the six items (including difficulties in interacting with client, feeling exhausted, wanting to reduce contact time, experiencing frustration, giving more but receiving less in return, and wanting to disengage from them). Each item was on a five-point scale: “never”, “not often”, “at times”, “often”, and “always”. The mean score of these six questions was calculated and then cutoff thresholds for client-related burnout were defined at the 90th percentile [29]. The 90th-percentile scores for the client-related burnout was 50.

### 2.4. Statistical Analyses

Demographics, socioeconomic characteristics, work conditions, workplace violence, and mental wellbeing of the study participants were described. Logistic regression analysis was used to estimate the odds ratio (OR) and 95% confidence interval (CI) for the effects of different types and sources of workplace violence on mental wellbeing, adjusted for sex, age, education level, marital status, annual income, work tenure, job control, and psychological job demands. Types and sources of workplace violence were mutually adjusted by including them in the same model for each outcome variable. In addition, the mediating effect of client-related burnout on the relationship between different source of workplace violence and poor mental health was evaluated using the ‘mediation' package in R software (v.4.4.0). This analysis decomposes the total effect of violence on poor mental health into direct and indirect effects, with the indirect effect representing mediation through client-related burnout. Chi-square tests were used to compare the differences in experiences of workplace violence, including types and sources, between work locations. The differential effect of violence on mental wellbeing in participants working in residential facilities or home settings was examined using simple effect analysis. Data analyses were performed using SAS 9.4 (SAS Institute Inc., Cary, NC, USA).

## 3. Results

The mean age of the participants were 46.42 years (standard deviation [SD] = 11.81), and 790 (84.31%) were women (Table 1). A total of 490 (52.41%) had a secondary education or lower. Regarding work locations, 72.15% provided services in a home setting. Regarding mental wellbeing, 239 (25.51%) reported poor mental health and 178 (19.00%) experienced high client-related burnout.

Verbal violence was the most prevalent type of workplace violence (26.04%), followed by physical violence (16.33%), psychological violence (15.69%), and sexual harassment (13.55%) (Table 2). In the adjusted logistic regression models, participants who had experienced psychological violence were more likely to have poor mental health (adjusted OR = 2.38, 95% CI = 1.40–4.05). In addition, those who had experienced verbal violence and sexual harassment had higher odds of experiencing client-related burnout (adjusted OR = 2.03 and 1.75, respectively).

**Figure 1:**
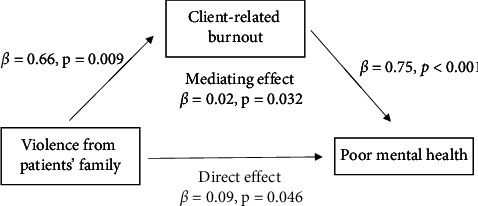
Mediating effect of client-related burnout on the relationship between violence from patients' families and poor mental health.

Violence from patients was the most prevalent source of workplace violence (26.04%), followed by violence from patients' family (11.21%), and colleagues or supervisors (5.98%) (Table 3). In the adjusted logistic regression models, participants who had experienced violence from patients' family and colleagues or supervisors were more likely to have poor mental health (adjusted OR = 1.78 and 2.32, respectively). In addition, those who had experienced violence from patients (adjusted OR = 1.65 and 95% CI = 1.12–2.43), patients' family (adjusted OR = 1.94 and 95% CI = 1.18–3.20), and colleagues or supervisors (adjusted OR = 2.01 and 95% CI = 1.07–3.76) were significantly more likely to have client-related burnout. To further investigate, we assessed the mediating effect of client-related burnout on the relationship between different source of the violence and poor mental health. The results showed that client-related burnout significantly mediated the association between violence from patients' families and poor mental health (*β* = 0.02 and *p*=0.032; Figure 1). However, client-related burnout did not mediate the association (*β* = 0.02 and *p*=0.068).

**Figure 2:**
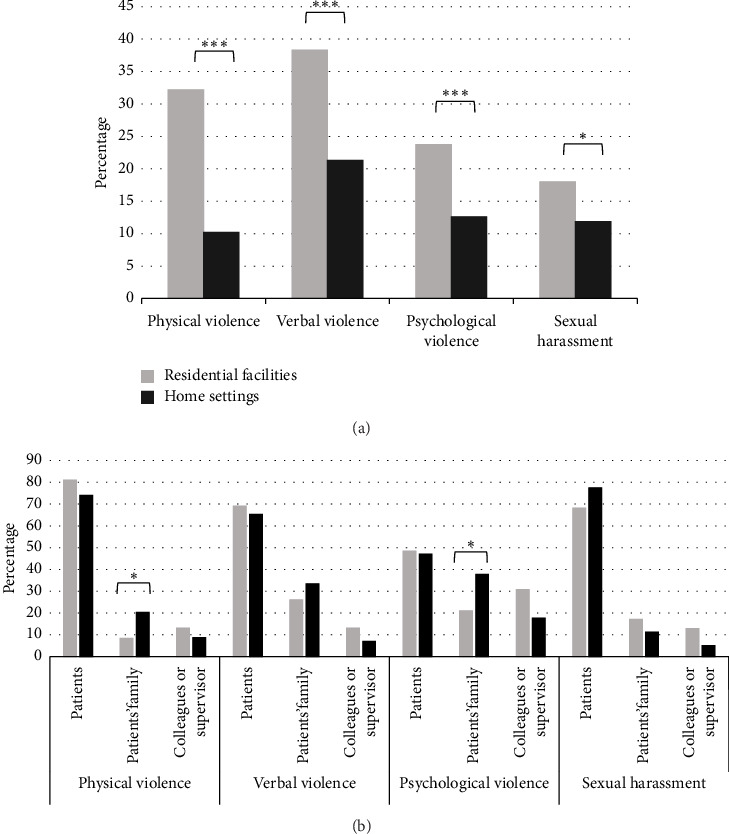
The distribution of four types of violence among all study participants (a) and source of violence among workplace violence victims (b) by work location. Differences between groups were examined using chi-square tests. ⁣^∗^*p* < 0.05, and ⁣^∗∗∗^*p* < 0.001.

Figure 2 illustrates the prevalence of workplace violence experiences among participants in different work locations. Compared with participants who provided services at home settings, those who provided services at residential facilities had a higher prevalence of all four types of violence (*p* values < 0.05). The difference between work locations was large for physical violence (32.18% vs. 10.21%) but small for sexual harassment (18.01% vs. 11.83%). A higher percentage of violence victims working at home settings experienced physical violence (20.29% vs. 8.33%, *p*=0.03) and psychological violence from patients' family (37.65% vs. 20.97%, *p*=0.03), compared with those working in residential facilities. The simple effect of workplace violence on mental wellbeing showed a stronger association of psychological violence with poor mental health, as well as verbal violence and sexual harassment with client-related burnout among participants working in home settings compared to residential facilities (Table 4).

## 4. Discussion

In this study, we investigated the influence of four types of workplace violence in different work locations on mental wellbeing among LTC-NAs. Our findings indicate that experiencing psychological violence may contribute to poor mental health, while experiencing verbal violence and sexual harassment is associated with client-related burnout. In addition, experiencing violence from patients' family and colleagues or supervisors may contribute to poor mental health and client-related burnout. Furthermore, client-related burnout was found to mediate the relationship between violence from patients' family and poor mental health. Workplace violence was more prevalent in residential facilities compared with home settings; however, their associations with mental wellbeing were more significant among LTC-NAs working in home settings.

Physical violence from patients with dementia in LTC settings is common and sometimes accepted as part of the job by LTC healthcare workers [3, 13]. In addition to physical violence, our findings observed that psychological violence and verbal violence are associated with adverse mental wellbeing among LTC-NAs. Previous studies have suggested that chronic exposure to nonphysical workplace violence increases the risk of developing mental disorders, burnout, and depression among healthcare workers [9, 30–32]. The observations in this study align with findings that nonphysical violence is more common and more strongly associated with poor mental health among hospital-working nurses [22]. Among LTC-NAs, psychological and verbal violence also involve racial/ethnic discrimination, as many LTC-NAs are immigrant workers belonging to minority ethnic groups in societies [13, 14], which could be pronounced in home settings. Sexual harassment is associated with client-related burnout in this study. In addition, female healthcare professionals are prone to experiencing burnout due to workplace sexual harassment [33]. While nonphysical violence experienced by LTC-NAs is often underreported, the high prevalence and significant impact on mental health warrant more attention.

Recent studies have focused on workplace violence within the context of LTC residential facilities [12–14]. However, this study found a novel paradox in the prevalence of workplace violence and its association with mental wellbeing among LTC-NAs working in residential facilities and home settings. It is unsurprising that physical violence was much more prevalent in residential facilities due to the higher disease severity of patients. However, the differences in the experience of sexual harassment were rather small between work locations, underscoring the prevalence of this type of workplace violence among LTC-NAs working in home settings [17]. The stronger association of nonphysical violence and sexual harassment with adverse mental health in home settings may result from the lack of environmental protective settings and having to deal with violence alone [18–20]. A lack of witnesses within home settings makes victims less willing to report and bring consequences to perpetrators [17, 34]. Support from colleagues and supervisors helps in coping with workplace violence [12, 35]; however, they are less timely available in home care settings [36].

Another explanation for the stronger association in LTC-NAs working in home settings could be that patients' families are a significant source of violence for LTC-NAs working in home settings, as observed in this study. Workplace violence from patients' families could have a more profound mental impact than violence from patients because healthcare workers tend to forgive patients with mental disabilities [37]. This is consistent with our observation that violence from patients were not associated with poor mental health, while violence from patients' family or colleagues and supervisors were. However, 64.5% of psychological violence to healthcare workers in hospitals was attributed to patients' family members [38]. Our finding also revealed that client-related burnout serves as a pathway through which experiencing violence from patients' family may lead to poor mental health. The lower perceived authority of homecare LTC-NAs as paraprofessional workers also results in their vulnerability to workplace violence in homecare settings [17]. Exploring how the interaction between LTC-NAs and patients' families, who are possibly informal caregivers bearing heavy caregiving burden, affects LTC-NAs' health requires further exploration. In addition, previous studies have shown that workplace violence from colleagues or supervisors has a more significant impact on mental health than violence from patients [22]. Such violence often involves sustained contact with perpetrators before and after incidents, resulting in heightened psychological and physical distress [39]. LTC-NAs reported frustration over insufficient administrative support, citing poor communication and a general lack of recognition of the violence they experience [13]. Further research is needed to develop effective strategies to strengthen organizational factors, such as safety culture and violence management procedures.

This study possesses the advantage of focusing on the growing population of LTC-NAs across diverse work locations. Furthermore, we differentiated types of workplace violence and employed validated questionnaires to evaluate and adjust for work conditions in the analyses. Nevertheless, this study has limitations. First, the cross-sectional design introduces the possibility of reverse causation. Participants with poor mental wellbeing may tend to recall or report more workplace violence experiences. We have excluded participants with clinical diagnosis of mental disorders to minimize the potential bias. Second, dependence on self-reported workplace violence experience entails the potential for subjectivity bias, influenced by participants' perceptions. Nevertheless, workplace violence is often underreported [38]; therefore, subjective reports of workplace violence may capture a more realistic picture than registered violence reports. Third, our study centered on active employees, potentially leading to an underestimation of the impacts of workplace violence, as individuals unable to cope with it might have already departed from their positions. Fourth, this study used convenience sampling, which may introduce selection bias and limit generalizability. Our study participants comprised 27.85% from residential facilities and 72.15% from home settings, differing slightly from the distribution of LTC-NAs in Taiwan (approximately 38% in residential institutions and 62% in home-based and community-based long-term care units) [1]. In addition, recruiting participants through training courses and workplace meetings may have overrepresented those more engaged in professional development or aware of workplace challenges. Therefore, our sample may not fully represent the broader LTC-NA population in Taiwan. Future studies should consider stratified sampling to improve representativeness. Lastly, certain factors related to mental health and client-related burnout, such as the health status of the care recipient, the caregivers' willingness to provide care, and individual personality traits, were not included in this study. These correlated factors should be incorporated into future research.

## 5. Conclusion

Workplace violence can negatively affect the mental wellbeing of LTC-NAs. Therefore, protective policies and organizational training programs should be tailored to ensure a sustainable LTC workforce [14]. Regulations mandating workplace violence prevention programs should be developed and enforced by policymakers, while employers are responsible for implementing comprehensive training and support systems to safeguard LTC-NAs. Notably, risk factors for workplace violence vary across different work locations. It is noteworthy that risk factors for workplace violence vary across different work locations. In home settings, violence is common when there are very close or very distant worker-client relationships and when care plans are not inclusive of clients' needs [40]. In residential facilities, not having enough time to assist residents with activities of daily living is associated with experiencing injuries from physical assaults [8]. Therefore, organizational strategies should be tailored according to the work context for LTC-NAs. For instance, regular discussions with supervisors about service recipients' needs, training in interaction skills with patients' families, and maintaining an appropriate distance with the patient are essential for LTC-NAs working in home settings. Regarding management support, effective communication with supervisors about customer and safety issues is crucial and beneficial in ensuring the safety of home care LTC-NAs [17, 35]. In addition, healthcare workers should be empowered to report violent incidents without fear of retaliation and be provided with resources for coping and recovery. In terms of care approaches, negotiating or persuading care recipients instead of using force and maintaining regular contact with them have been shown to decrease violence from patients with mental illness [20]. It is also imperative to train LTC-NAs to recognize triggers of violent behavior [13]. Ultimately, workplace violence should not be tolerated or accepted as part of LTC-NAs' work. The fundamental goal for LTC workplaces should be to be free of violence, ensuring a safe and decent working environment. Future research is needed to examine the long-term effects of workplace violence interventions and the role of organizational culture in mitigating violence. In addition, it is essential to explore innovative strategies for preventing workplace violence across diverse healthcare settings.

## Figures and Tables

**Table 1:** Demographics characteristics, work conditions, and mental health of the study sample (*N* = 937).

Variables	Mean	SD
Demographic characteristics		
Age (year)	46.42	11.81

	***N***	**%**

Sex (female)	790	84.31
Education		
Secondary or lower	490	52.41
University or above	445	47.59
Marital status		
Married	566	60.41
Other status	371	39.59
Annual income		
High	125	13.34
Low	812	86.66
Work conditions		
Work location		
Residential facilities	261	27.85
Home settings	676	72.15
Work tenure		
≤ 1 year	261	27.85
2-3 years	292	31.16
≥ 4 years	384	40.98
Job control		
High	500	53.36
Low	437	46.64
Psychological job demands		
High	454	48.45
Low	483	51.55
Mental health		
Brief Symptom Rating Scale (BSRS)		
Good mental health (< 6)	698	74.49
Poor mental health (≥ 6)	239	25.51
Client-related burnout		
< 50	759	81.00
≥ 50	178	19.00

Abbreviation: SD = standard deviation.

**Table 2:** Distribution of four types of violence and adjusted odds ratios (ORs) and 95% confidence intervals (CIs) for poor mental health and client-related burnout (*N* = 937).

Type of violence	*N* (%)	Poor mental health	Client-related burnout
		OR (95% CI)	OR (95% CI)
*Physical violence*			
No	784 (83.67)	1	1
Yes	153 (16.33)	1.23 (0.78–1.96)	1.38 (0.84–2.27)

*Verbal violence*			
No	693 (73.96)	1	1
Yes	244 (26.04)	0.97 (0.62–1.53)	2.03 (1.25–3.29)⁣^∗^

*Psychological violence*			
No	790 (84.31)	1	1
Yes	147 (15.69)	2.38 (1.40–4.05)⁣^∗∗^	0.81 (0.45–1.44)

*Sexual harassment*			
No	810 (86.45)	1	1
Yes	127 (13.55)	1.07 (0.64–1.78)	1.75 (1.001–3.05)⁣^∗∗^

*Note:* The logistic regression models were adjusted for sex, age, marital status, education level, annual income, work tenure, job control, psychological job demands, and mutually for the type of violence.

⁣^∗^*p* < 0.05.

⁣^∗∗^*p* < 0.01.

**Table 3:** Distribution of violence sources and adjusted odds ratios (ORs) with 95% confidence intervals (CIs) for poor mental health and client-related burnout (*N* = 937).

Source of the violence	*N* (%)	Poor mental health	Client-related burnout
		OR (95% CI)	OR (95% CI)
*Patients*			
No	693 (73.96)	1	1
Yes	244 (26.04)	1.10 (0.77–1.57)	1.65 (1.12–2.43)⁣^∗^

*Patients' family*			
No	832 (88.79)	1	1
Yes	105 (11.21)	1.78 (1.13–2.82)⁣^∗^	1.94 (1.18–3.20)⁣^∗∗^

*Colleagues or supervisors*			
No	881 (94.02)	1	1
Yes	56 (5.98)	2.32 (1.30–4.17)⁣^∗∗^	2.01 (1.07–3.76)⁣^∗^

*Note:* The adjusted logistic regression models were adjusted for sex, age, marital status, education level, annual income, work tenure, job control, psychological job demands, and mutually for the source of violence.

⁣^∗^*p* < 0.05.

⁣^∗∗^*p* < 0.01.

⁣^∗∗∗^*p* < 0.001.

**Table 4:** Simple effect of four types of violence for poor mental health and client-related burnout in adjusted logistic regression models at different work location (*N* = 937).

	Outcome: poor mental health	
	Residential facilities (*N* = 261)	Home settings (*N* = 676)
	OR (95% CI)	OR (95% CI)
Physical violence	1.71 (0.89–3.26)	1.21 (0.65–2.23)
Verbal violence	0.89 (0.45–1.77)	1.05 (0.63–1.75)
Psychological violence	2.07 (1.01–4.24)⁣^∗^	2.67 (1.43–4.97)⁣^∗∗∗^
Sexual harassment	0.99 (0.46–2.15)	1.06 (0.58–1.93)

	**Outcome: client-related burnout**	
	**Residential facilities (*N* = 261)**	**Home settings (*N* = 676)**
	**OR (95% CI)**	**OR (95% CI)**

Physical violence	0.92 (0.48–1.75)	1.63 (0.82–3.25)
Verbal violence	1.51 (0.78–2.92)	2.35 (1.31–4.21)⁣^∗∗^
Psychological violence	0.54 (0.26–1.12)	1.16 (0.58–2.34)
Sexual harassment	1.30 (0.60–2.82)	2.37 (1.22–4.61)⁣^∗^

*Note:* Models were adjusted for sex, age, marital status, education level, annual income, work tenure, job control, psychological job demands, and other types of violence mutually.

⁣^∗^*p* < 0.05.

⁣^∗∗^*p* < 0.01.

⁣^∗∗∗^*p* < 0.001.

## Data Availability

The data that support the findings of this study are available from the corresponding author upon reasonable request.
